# Efficacy of Acupuncture in Reducing Preoperative Anxiety: A Meta-Analysis

**DOI:** 10.1155/2014/850367

**Published:** 2014-09-02

**Authors:** Hyojeong Bae, Hyunsu Bae, Byung-Il Min, Seunghun Cho

**Affiliations:** ^1^Department of East-West Medicine, Graduate School, Kyung Hee University, Hoegi-Dong No. 1, Dongdaemun-Gu, Seoul 130-701, Republic of Korea; ^2^Soram Korean Medicine Hospital M Tower Building, Samsung-Dong No. 154-11, Gangnam-Gu, Seoul 135-879, Republic of Korea; ^3^Department of Physiology, College of Korean Medicine, Kyung Hee University, Hoegi-Dong No. 1, Dongdaemun-Gu, Seoul 130-701, Republic of Korea; ^4^Department of Physiology, College of Medicine, Kyung Hee University, Hoegi-Dong No. 1, Dongdaemun-Gu, Seoul 130-701, Republic of Korea; ^5^Hospital of Korean Medicine, Kyung Hee University Medical Center, Hoegi-Dong No. 1, Dongdaemun-Gu, Seoul 130-701, Republic of Korea

## Abstract

*Background*. Acupuncture has been shown to reduce preoperative anxiety in several previous randomized controlled trials (RCTs). In order to assess the preoperative anxiolytic efficacy of acupuncture therapy, this study conducted a meta-analysis of an array of appropriate studies. *Methods*. Four electronic databases (MEDLINE, EMBASE, CENTRAL, and CINAHL) were searched up to February 2014. In the meta-analysis data were included from RCT studies in which groups receiving preoperative acupuncture treatment were compared with control groups receiving a placebo for anxiety. *Results*. Fourteen publications (*N* = 1,034) were included. Six publications, using the State-Trait Anxiety Inventory-State (STAI-S), reported that acupuncture interventions led to greater reductions in preoperative anxiety relative to sham acupuncture (mean difference = 5.63, *P* < .00001, 95% CI [4.14, 7.11]). Further eight publications, employing visual analogue scales (VAS), also indicated significant differences in preoperative anxiety amelioration between acupuncture and sham acupuncture (mean difference = 19.23, *P* < .00001, 95% CI [16.34, 22.12]). *Conclusions*. Acupuncture therapy aiming at reducing preoperative anxiety has a statistically significant effect relative to placebo or nontreatment conditions. Well-designed and rigorous studies that employ large sample sizes are necessary to corroborate this finding.

## 1. Introduction

Anxiety prior to undergoing surgery is experienced by approximately 60–70% of adult patients [1]. The effects of reducing preoperative anxiety can be observed by estimating heart rate (HR), blood pressure (BP), and neuroendocrinological changes [2]. These effects can also be determined during or after surgery through the examination of analgesic requirements, behavioral recovery, time taken to awaken, pain, and whether such outcomes also engender additional financial costs to patients [3, 4]. Pharmacological (e.g., opioids and sedatives used as anxiolytics) and psychological interventions (e.g., music and preparatory education regarding the operation) are commonly used to reduce preoperative anxiety [5, 6]. However, conventional medical treatments are only moderately effective and often produce problematic side effects, including bradycardia, hypotension, drowsiness, respiratory depression, pruritus, laryngeal rigidity, postoperative nausea and vomiting (PONV), delayed emergence, and tolerance and dependence, thereby prolonging patient recovery and treatment duration [7, 8]. Therefore, there is a clear need for more effective, safer interventions. This has led to an increase in the attention received by complementary and alternative interventions such as acupuncture, which is the most widely used of such approaches [9]. Patients benefit from the lack of side effects and relatively low cost involved in acupuncture [10].

Acupuncture is gaining popularity in western medical culture as a tool for pain relief [11, 12], and evidence is emerging concerning its potential mechanisms of action. For example, electroacupuncture blocks pain by activating a variety of bioactive chemicals via peripheral, spinal, and supraspinal mechanisms [13].

Recently, several studies have evaluated the “extra 1” acupuncture or acupressure point with respect to relieving preoperative and general anxiety [8, 14–18]. However, to date, there have been no meta-analyses performed regarding this topic; therefore, we sought to summarize and critically assess evidence from randomized controlled trials (RCTs). The aim of this meta-analysis was to evaluate the efficacy of various types of acupuncture therapy with respect to reducing preoperative anxiety.

## 2. Methods

A meta-analysis of the literature was conducted according to the “Preferred Reporting Items for Systematic Reviews and Meta-Analyses” (PRISMA) statement pertaining to reporting systematic reviews and meta-analyses of studies that evaluate preoperative care interventions.

### 2.1. Literature Search

Electronic searches were performed independently by two authors on MEDLINE (1950 to February 2014), Embase (1980 to February 2014), CENTRAL (the Cochrane Library 2014, Issue 1), and CINAHL (1982 to February 2014). As all of these databases employ their own subject headings, each was searched independently. We did not restrict our search on the basis of language, publication type, or year. Article bibliographies were checked for current relevant publications and experts in the field contacted. We also searched for additional relevant journals that may have been overlooked in the initial electronic search and available proceedings of conferences for information on additional trials. In an effort to identify other published, unpublished, and ongoing relevant researches, we also searched the reference sections of pertinent studies.

Keywords used to search for the RCTs were (anxiety OR anxioly∗ OR sedat∗ OR distress OR fear OR panic OR stress, psychological OR stress, physiological) AND (acupressure OR acupoint OR auriculotherapy OR meridians OR electroacupuncture OR acupuncture) AND (surgical OR procedure∗ OR preoperative care OR surgery) AND (randomized controlled trial [PT] OR randomized [AB] OR controlled clinical trial [PT] OR placebo [AB] OR clinical trial as topic [SH] OR randomly [AB] OR trial [TI]) in MEDLINE. Each database used its own subheadings and was searched individually.

The exclusion and inclusion criteria were applied separately by the two authors, who scanned the titles and abstracts of each record retrieved from the search. If information in the abstract clearly indicated that the trial did not meet our requirements, it was rejected. When a title or abstract could not be rejected with certainty, the authors inspected the full text independently and applied an inclusion criterion form to definitively assess its eligibility. Where disagreements occurred, the authors discussed the issue until a consensus was reached. If an article was excluded, a record was of the reason for exclusion. The final step was to exclude double publications.

### 2.2. Study Types

The meta-analysis included studies on inpatients and outpatients and nonemergency, emergency, and transported patients, who were scheduled to undergo both major and minor surgical or endoscopic procedures. Dental surgery procedures were also included. No restrictions were placed on age, sex, or ethnicity, but patients were excluded if they had a history of psychiatric or neurological problems or serious medical conditions, such as abuse of or addiction to drugs or alcohol, or used analgesics within the week preceding the procedure.

Included studies were restricted to RCTs that compared all forms of acupuncture-treated (delivered using classical sterile single-use needles, plastic balls, or occlusive press needles) and control groups, which included nontreatment or placebo treatment (sham acupuncture unrelated to known acupoints for treatment, using a superficial depth of acupuncture, or without electronic stimulation), with the aim of reducing preoperative anxiety. Quasirandomized trials were not included. No restrictions were imposed with respect to publication type or language.

We did not include studies in which treatments were administered on days other than the day of surgery. The primary outcome was the degree of reduction in preoperative anxiety produced by acupuncture in controlled trials involving a group to whom acupuncture was administered and a control group. Measures of anxiety included the State Anxiety Subscale (STAI-S) of the State-Trait Anxiety Inventory (STAI), which asks respondents how they feel “right now” on 20 items measuring subjective feelings of apprehension, tension, nervousness, worry, and activation/arousal of the autonomic nervous system. Anxiety scores in the STAI-S range from 1 (*not at all*) to 4 (*very much so*) for each item [26]. The mean difference (MD) in changes in continuous scale scores for preoperative anxiety represented a degree of reduction in STAI and visual analogue scale (VAS) scores. VAS simply indicated levels of anxiety according to a 100 mm scale line, where 0 represents a complete absence of anxiety and 100 the greatest possible level of anxiety. Where scales were scored between 0 and 10, values and standard deviations were multiplied by a factor of 10 [27]. Secondary outcomes included physiological variables, heart rate (HR), bispectral index (BIS), and blood pressure (BP), patient satisfaction, and adverse events.

#### 2.2.1. Quality Assessment

The two authors assessed all included studies for risk of bias and were blinded to each other's assessments. Continuous data were preferred to binary data because most of the eligible studies reported continuous outcomes. Further information was requested from the authors where articles contained inadequate information to make a decision about eligibility. Quality assessment for all studies was undertaken according to the Cochrane Handbook for Systematic Reviews of Interventions [28]. Studies were assessed by reviewers drawn from six domains. If articles contained inadequate information to allow for a decision made about their eligibility, then further information was requested from the authors. No studies were excluded from the analysis as a result of the quality assessment procedure.

#### 2.2.2. Data Synthesis and Statistical Analyses

Continuous data were summarized as mean differences (MD) between pre- and posttreatment STAI-S or VAS scores. The degree of reduction in preoperative anxiety, with 95% confidence intervals (CIs), was calculated using Review Manager (RevMan) software (version 5.2 for Windows, The Nordic Cochrane Centre, Copenhagen, Denmark). If the 95% CI included a value of 0, then no significant difference existed between acupuncture-treated and control groups.

We subtracted final values from baseline mean values, even if these were not presented explicitly, such that a positive MD of the changes in scores indicated effective reduction of preoperative anxiety. If either of the standard deviations (at baseline or final) was unavailable, then one was substituted for the other if it was reasonable to assume that the intervention did not alter the variability of the outcome measure [28]:

(1) $$\begin{array}{c} \text{SD}=\sqrt{\left({{\text{SD}}_{1}}^{2}+\;{{\text{SD}}_{2}}^{2}-{2R}_{\text{corr}}\;{\text{SD}}_{1}{\text{SD}}_{2}\right)\;}. \end{array}$$

We considered a 30% greater reduction in STAI and VAS scores following acupuncture treatment, relative control conditions, to be clinically relevant [29, 30]. Our meta-analysis employed a random-effects model, which assumes that effects estimated across different studies are not identical. If there was significant heterogeneity, however, then a fixed-effects model was applied. Concerning statistically significant differences in side effects, “number needed to harm” (NNH) values were calculated. Forest plots were used to graphically represent and evaluate treatment effects. Funnel plots of effects estimates against standard error were generated if a sufficient number of studies for each treatment regimen was available [31].

A sensitivity analysis was performed in order to identify sources of heterogeneity and ensure the stability of results. We excluded studies with two or more unclear biases or a high risk of bias for any of the risks in key bias domains. An additional sensitivity analysis was performed where sample sizes exceeded 100.

Studies were combined in instances where statistical heterogeneity was not evident. Heterogeneity was examined via the *I* ^2^-test, where *I* ^2^ values of 50% or more were indicative of significant heterogeneity.

## 3. Results

### 3.1. Study Description

**Figure 1:**
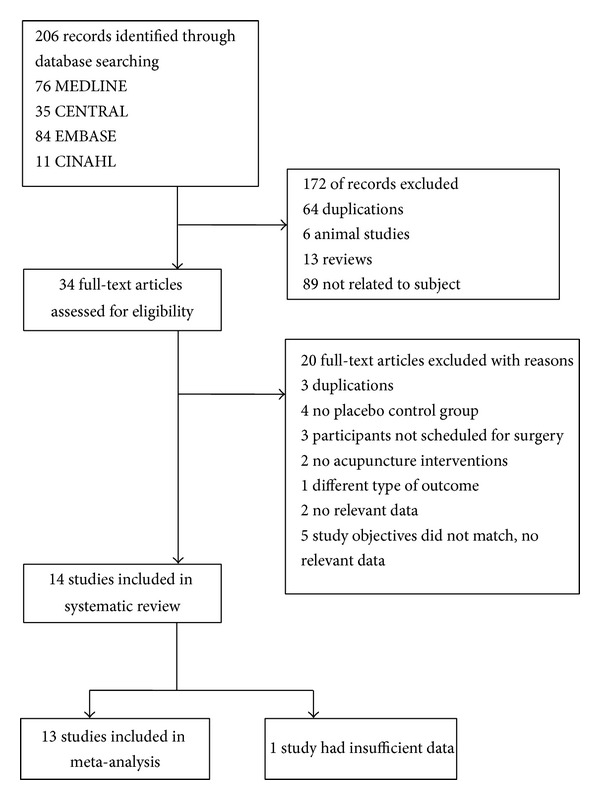
Flow chart for included studies.

An initial search identified 206 potentially relevant articles, of which 14 (*N* = 1,034) met our inclusion criteria and were thus added to the final analysis. The agreement rate, as measured using Cohen's kappa, was 0.9 [32]. Acupuncture treatments were administered to 439 patients; the other 595 participants served as controls. One author requested additional data from the authors of four studies; however, the data from one study were not obtained (Figure 1).

Studies offered acupuncture sessions lasting between 10 and 30 min; sessions were conducted in operating waiting rooms on the day of surgery. Two studies offered sessions during ambulance transfer [15, 22]. Participants were inpatients in one study and outpatients in two studies; the status of the participants in the remaining studies was unclear. Administration of acupuncture was examined during transportation and emergency cases in two studies and in nonemergency cases in eight studies; the environment in which acupuncture was administered was unclear in the remaining studies. Eight studies used acupuncture needles [10, 14, 15, 19–22, 24]; the other six used acupressure balls or beads [8, 16–18, 23, 25]. Five studies applied auricular acupoints, five others applied body acupoints, and four applied both. According to “Standards for Reporting Interventions in Clinical Trials of Acupuncture” (STRICTA), eight of the included studies reported the types of needles used, including the diameter and length as well as the manufacturer and/or the material, and the others reported only the types of needles. All of the studies were based on acupuncture point selection in traditional acupuncture theory. Various acupoints were used for decreasing preoperative anxiety in the included RCTs; the third eye (Yin-Tang), located between the two eyebrows, was commonly used in six trials, and the relaxation auricular point, located in the superior lateral wall of the triangular fossa, was also used in six trials. Needle stimulation was administered manually in four RCTs and electronically (2 Hz 25 V) in one RCT. Two studies reported “de qi” sensations, where reportage of such was recommended. These data are reported in the STRICTA recommendations [33]. Characteristics of all included studies are provided in Table 1.

**Figure 2:**
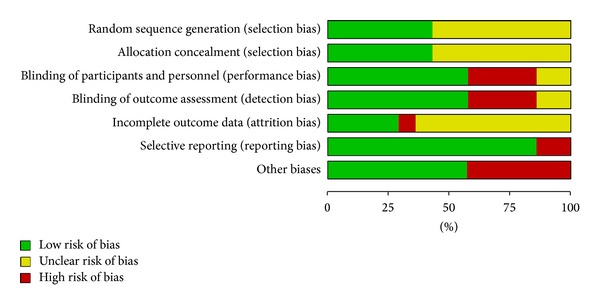
Risk of bias. Each risk of bias item presented as percentages across all included studies.

**Figure 3:**
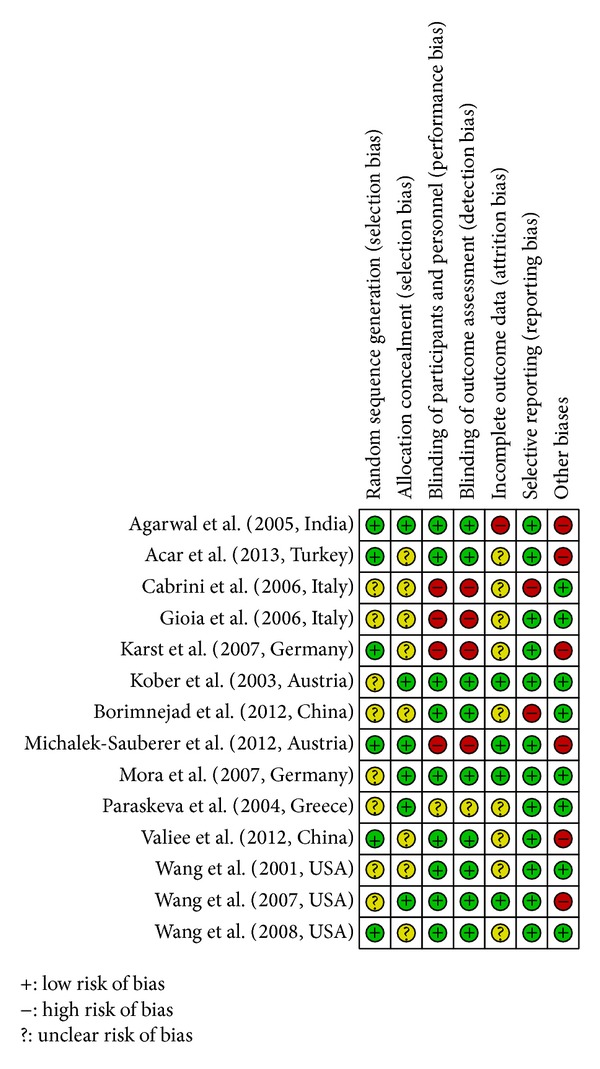
Methodological quality summary. Methodological quality indices for all included studies. “+” = low risk of bias, “−” = high risk of bias, and “?” = unclear risk of bias.

The 14 included studies exhibited various degrees of bias susceptibility (Figures 2 and 3). The agreement rate, as measured using Cohen's kappa, was 0.8 [32]. Only six studies reported concealed allocation; the other six described a method of adequate randomization, although the word “randomization” appeared in all of the articles. Thirteen studies prevented blinding of the participants. Participants in these studies had no previous experience of acupuncture. According to STRICTA, two studies enquired after patients' beliefs as a group: there were no significant differences [20, 24].

### 3.2. STAI-S

**Figure 4:**
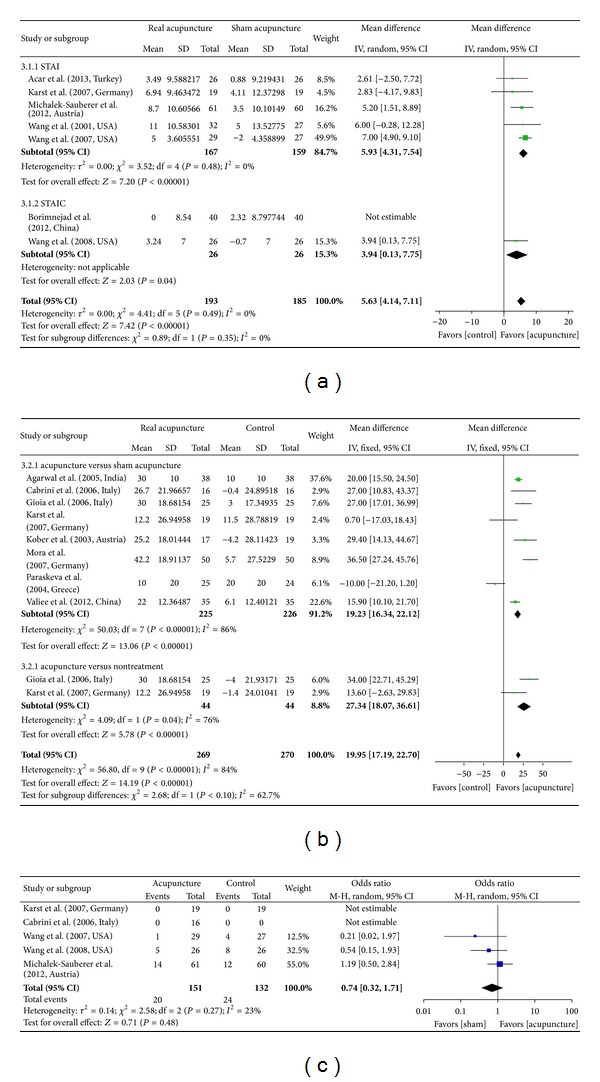
Forest plot of acupuncture efficacy in reducing preoperative anxiety. (a) STAI acupuncture versus sham acupuncture. (b) VAS acupuncture versus control groups. (c) Side effect acupuncture versus sham acupuncture. The term “STAIC” in part (a) indicates the State Anxiety Subscale of the State-Trait Anxiety Inventory in children. The term “events” in part (c) indicates the number of patients who reported adverse events including PONV. “Weight” refers to the contribution of each study to the side effects total.

A meta-analysis of six studies using the STAI-S to examine state anxiety in 378 participants revealed significantly lower state anxiety levels in participants who received real versus sham acupuncture interventions (MD = 5.63, *P* < .00001, 95% CI [4.14, 7.11], Figure 4(a)). This was expressed in mean group differences in pre- and postintervention STAI-S scores. A random-effects model was used in the analysis, and statistical heterogeneity was not observed across the studies (*I* ^2^ = 0%). Regarding studies distinguishing between adults and children, a significant reduction in scores was observed in five studies that measured STAI-S scores in adults (MD = 5.93, *P* < .00001, 95% CI [4.31, 7.54]). Similarly, a significant reduction was found in one study measuring STAI-S scores in children (STAI-C, MD = 3.94, *P* = .04, 95% CI [0.13, 7.75]). The width of the CI and the *P* value suggested that these data were statistically sufficient to allow for a conclusion; however, the reduction in the mean change in STAI-S scores did not reach clinical significance [34, 35].

When restricting the analysis to studies with 100 or more participants, acupuncture treatment was still associated with significantly decreased preoperative anxiety [24] (MD = 5.2, *P* = .006, 95% CI [1.51, 8.89]). A sensitivity analysis, which removes studies with lower-quality methodologies, was not performed for any of the included studies.

### 3.3. VAS

We identified eight studies (*n* = 495) that employed VAS measurements. The pooled analysis demonstrated that acupuncture interventions led to greater reductions in VAS anxiety relative to sham acupuncture (MD = 19.23, *P* < .00001, 95% CI [16.34, 22.12], Figure 4(b)). A fixed-effects model was used owing to the heterogeneity of the results (*I* ^2^ = 86%). Two studies reported significant decreases in preoperative anxiety following acupuncture treatment versus nontreatment (MD = 27.34, *P* < .00001, 95% CI [18.07, 36.61]). These data were statistically significant, based on the *P* value and the width of the CI, and the mean difference was closer to clinical significance in the acupuncture-treated group relative to the control group; however, the sample size was small (*n* = 88). A sensitivity analysis was performed for two of the included studies [23, 25] in order to investigate the source of their heterogeneity. Acupuncture's association with reduced preoperative anxiety, in comparison to sham acupuncture, remained in place (MD = 34.59, *P* < .00001, 95% CI [26.68, 42.51]) following the exclusion of studies with lower-quality methodologies, where this exclusion also improved the homogeneity of results (*I* ^2^ = 0%). Although the MD was based on more than 30 VAS change scores, it should not be considered conclusive in light of the small sample size (*n* = 136).

### 3.4. Subgroup Analysis

**Figure 5:**
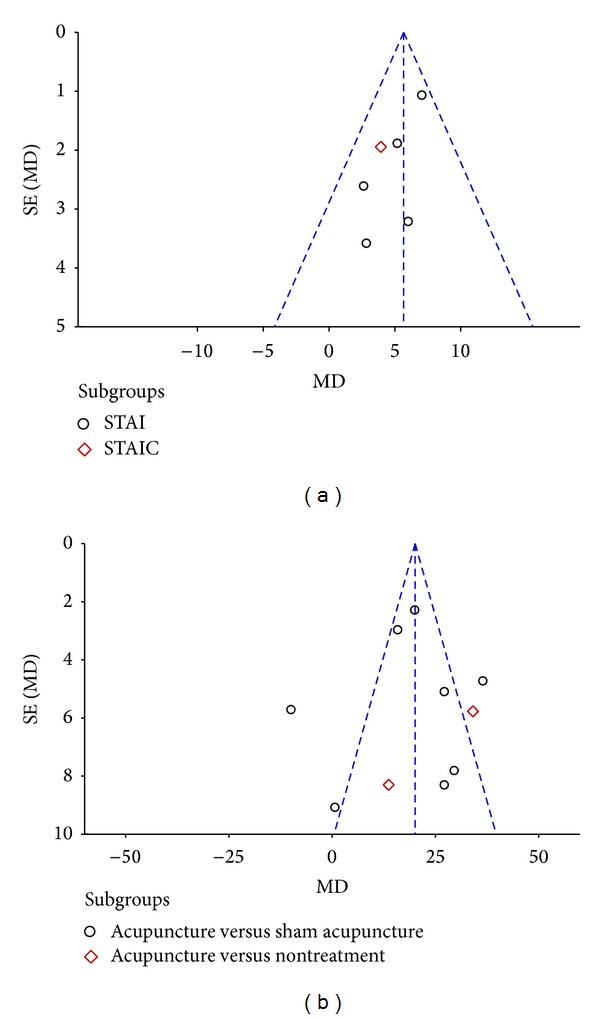
Funnel plot of the mean difference (MD) in anxiety ratings between acupuncture treatment and control groups, versus standard error (SE).

For both types of acupuncture instrument (needles and beads), acupoint location (body versus ear) had no impact on the primary outcome measure of preoperative anxiety. Publication bias was reported via Begg's funnel plot (Figure 5), where asymmetry of the plots may have arisen through publication bias and the relationship between trial size and effect size.

### 3.5. Secondary Outcomes

For exploratory purposes, additional analyses of secondary outcomes were performed for physiological variables (HR, BIS, and BP). Six studies measured heart rate before and after intervention; none of these reported a significant difference between the acupuncture and sham groups [10, 21–23, 25, 36]. Two studies also reported no significant difference in blood pressure [17, 23]. No significant changes in BIS scores were observed between groups in four studies [8, 14, 15, 18]; one of these also reported that BIS values did not differ between the groups before, during, or after acupuncture, but, during acupuncture, BIS scores were significantly lower in the group receiving acupuncture but not in the placebo group [15]. In contrast to the significant reductions seen for the primary outcome measure of anxiety, no significant difference in physiological measurements was identified.

### 3.6. Side Effects

Among studies reporting adverse events, two found no adverse events in either the acupuncture or sham acupuncture groups, relative to the control group, for which a burning sensation in response to intranasal medication was reported in 32.6% of the participants (NNH = 7) [10, 22]. Two RCTs reported PONV in both the intervention and control groups, but with no significant differences in rate of occurrence (OR = 0.42, *P* = 0.13, 95% CI [0.14, 1.29]) [18, 20]. Ear warmth and peculiar sensations and dizziness were reported in only one study, but there was no significant difference in occurrence rates between groups (Figure 4(c)) [24].

### 3.7. Patient Satisfaction

Two of the included studies investigated patient satisfaction via VAS scales (0–10 points) [20] and discontinuous numeric scales (from 1 to 5) [10]; no significant group differences were observed (MD = 0.38, *P* = .31, 95% CI [−0.35, 1.12]).

**Figure 6:**
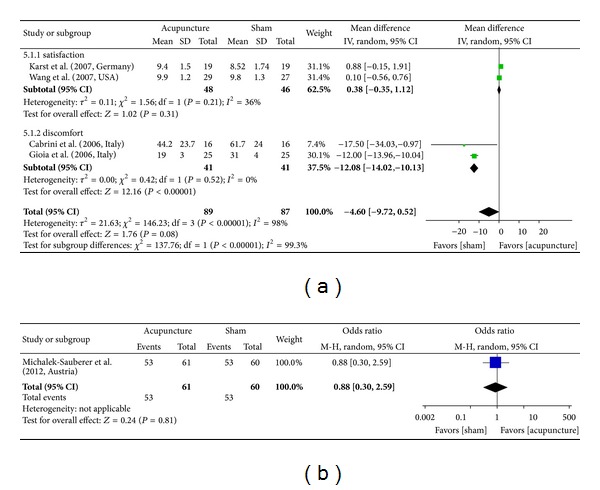
Forest plot depicting various outcomes for postsurgical patient satisfaction. (a) VAS (satisfaction and discomfort after surgery). (b) Number of patients reporting a “good” level of treatment satisfaction.

Another study investigated the comfort level associated with acupuncture treatment according to a dichotomous scale comprising “good” or “other” ratings; again, there were no significant differences (OR = 0.88, *P* = .81, 95% CI [0.30, 2.59]) [24]. Two other studies investigated discomfort according to VAS scale ratings (0–100 points) and reported that discomfort was higher in control groups (MD = −12.08, *P* < .00001, 95% CI [−14.2, − 10.13], Figure 6) [21, 22].

## 4. Discussion

This meta-analysis demonstrates that acupuncture therapy, administered in isolation, can decrease preoperative anxiety in patients with scheduled surgery. To our knowledge, there have been no other systematic reviews or meta-analyses of RCTs conducted concerning acupuncture's efficacy in reducing preoperative anxiety. Moreover, no restrictions were applied for age or language, and several literature databases were searched via a comprehensive strategy. A previous meta-analysis indicated that acupuncture treatment reduces postoperative pain and is associated with a lower incidence of nausea among PONV cases [37]. However, the sample was restricted to adults and there was wide variability in the type and timing of acupuncture regimens applied and the duration and number of treatment sessions.

Acupuncture was generally associated with greater reductions in anxiety prior to surgery relative to control (nontreatment) and sham treatment conditions. Based on the findings of the current meta-analysis, all varieties of acupuncture therapy, delivered in isolation to patients on the day of surgery, are effective.

Karst et al. [10] compared the effects of pharmaceutical agents and acupuncture for preoperative stress. They concluded that, although the number of studies included was insufficient for meaningful analysis, auricular acupuncture and intranasal midazolam were similarly effective for the treatment of anxiety.

Griffiths et al. [38] assessed the efficacy of interventions (pharmacological and nonpharmacological, including acupressure therapy) aiming to prevent nausea and vomiting in women undergoing regional anesthesia for a caesarean section. Acupressure was only found to be effective for intraoperative nausea and was not effective for postoperative nausea or vomiting. Their review was specifically concerned with pregnancy-related underlying risk factors for nausea and vomiting.

Some reviews have reported on studies involving infants and children. Several studies found no significant statistical or clinical differences in the efficacy of nonpharmacological methods, such as parental acupuncture versus sedative premedications [39–41]. The effects of parental acupuncture on children's anxiety remain unclear and were not evaluated in this study. Assuming that acupuncture reduces preoperative anxiety, the potential mechanisms of action may be similar to those previously documented for acupuncture [42, 43]. In our meta-analysis, two studies included participants under the age of 18, for whom the STAI-C, which was used in both studies, is considered the gold standard in the assessment of anxiety in children older than 6 years of age. This questionnaire is well validated, has been used in more than 1,000 studies [44], is easy to read, and can be administrated verbally. Although there are no data regarding the issue of clinical significance in the pediatric anxiety literature, we found that a minimum difference of 10% in state anxiety levels, as assessed by the adult version of the STAI-S, is considered clinically significant [34, 35]. Borimnejad et al. [16] reported significant differences not for an acupuncture-treated group but for a sham treatment group.

The present review has several limitations. The small number of included trials did not allow for the performance of a metaregression examining all of the possible predictors together, given the suggested threshold of 14 studies required per predictor [28]. The small number of included studies also resulted in wide CIs for the pooled results of many of the reported outcomes, thereby rendering the drawing of definitive conclusions difficult. In addition, we could not combine all of the results of the STAI-S in children owing to insufficient data, where postintervention anxiety scores in acupuncture treatment groups were occasionally not provided; in some instances, attempts to contact authors were also unsuccessful. Despite a general lack of relevant data, we did not exclude data in an effort to avoid publication bias.

Even when considering the caveats described above, our analyses support the possibility that acupuncture treatment is able to reduce preoperative anxiety better than sham acupuncture. Clinically important differences were observed in the reduction of preoperative anxiety between acupuncture-treated and control (nontreatment) conditions, although the overall sample size was small. The findings of our analyses are clinically important, in which the results support the proposition that acupuncture is beneficial in reducing preoperative anxiety. Based on this assumption, potential mechanisms of action may be similar to those documented in the acupuncture literature [42, 45].

Our study has identified some areas in which further research on acupuncture treatment is warranted. For example, it remains unclear as to whether there is a difference in the efficacy of acupuncture therapy versus conventional premedication treatments. Additional studies are also required in order to establish objective assessment methods and ideal techniques for blinding.

## 5. Conclusion

In conclusion, this meta-analysis suggests that acupuncture therapy aiming at reducing preoperative anxiety has some beneficial effects as compared to placebo or nontreatment alternatives. Further RCTs should be conducted to gain a better understanding of the role of acupuncture in this context.

## Figures and Tables

**Table 1:** Characteristics of included studies.

Author (year, location)	Subject age (years)	Number of participants (Acu^a^/Sham^b^/Con^c^1/Con2)	Surgery	Type of design	Type of intervention (duration, side, and type of stimulation)	Treated acupoints	Type of control group	Outcome measure reported (*P*value)	Adverse events reported (*n*)
Wang et al. (2001, USA) [19]	19–66	91 (31/32/27)	Elective ambulatory surgery (orthopedic, gynecologic, genitourinary, otolaryngologic, plastic, general ophthalmologic)	RCT^d^	Auricular acupressure needle (30 min, nondominant hand side)	Relaxation Tranquilizer Mmaster cerebral	(1) Traditional Chinese medicine group (2) Sham acupuncture	STAI^e^ (.01)	NR^f^

Wang et al. (2007, USA) [20]	18–65	56 (29/27)	Elective lithotripsy procedure	RCT	Auricular acupressure needle (dominant, 30 min) Bilateral body acupuncture with 2 Hz, 25 V electrical stimulation	Relaxation valium, master cerebral, ^k^LI4, ^l^LV3	(1) Sham auricular acupressure, no electrical stimulation, superficial insertion in the same locations	STAI (.029)	PONV^g^-acu (4%) con (15%) *P* = .412

Wang et al. (2008, USA) [18]	8–17	52 (26/26)	General anesthesia for GI endoscopy (upper endoscopy and colonoscopy)	RCT	Acupressure beads (30 min)	Yin Tang	(1) Sham acupressure	STAIC (.012)	PONV-acu (5) con (8)

Paraskeva et al. (2004, Greece) [15]	NR	49 (25/24)	Minor or moderate surgery	RCT	Acupuncture (15 min)	Yin Tang	(1) Sham acupuncture	VSS^h^ (NS)	NR

Gioia et al. (2006, Italy) [21]	71.3 (mean age)	75 (25/25/25)	Cataract surgery under topical anesthesia	RCT	Body acupuncture (20 min, dominant) Auricular (manually rotated De Qi) acupuncture	LI4, LV3 ^m^PC6, ^n^HT7 ^o^TE5 Shenmen	(1) Nontreatment (2) Sham acupuncture	VAS^i^ (.037)	NR

Cabrini et al. (2006, Italy) [22]	18+	48 (16/16/16)	Elective diagnostic fiberoptic bronchoscopy	RCT	Bilateral body acupuncture and auricular (20 min, manually rotated De Qi) acupuncture	^ p^LU7, PC6 LI4, HT7 Shenmen	(1) Nontreatment (2) Sham acupuncture	VAS (.002)	None

Karst et al. (2007, German) [10]	18–65	67 (19/19/19/10)	Dental extractions	RCT	Acupuncture (nondominant, 25 min)	Relaxation Tranquilizer Master cerebral	(1) Placebo auricular acupuncture (2) Intranasal midazolam (3) Nontreatment	STAI (<.001) VAS (.012)	Nasal burning (7) None in the other groups

Mora et al. (2007, German) [23]	65–90	100 (50/50)	Transported by ambulance before receiving ESWL	RCT	Bilateral auricular acupressure (NR, 1 mm plastic ball)	Relaxation	(1) Sham acupressure	VAS (.001)	NR

Michalek-Sauberer et al. (2012, Austria) [24]	18+	182 (61/60/61)	Dental treatment	RCT	Auricular acupuncture (20 min, dominant)	Relaxation Tranquilizer Master cerebral	(1) Sham acupuncture (2) Nontreatment	STAI (.008)	Acupuncture (14), Sham (12)

Acar et al. (2013, Turkey) [14]	18–65	52 (26/26)	General/regional anesthesia	RCT	Auricular acupuncture (20 min, ear-press needle)	Yin Tang	(1) Sham acupressure	STAI (<.05)	NR

Kober et al. (2003, Austria) [25]	23–89	36 (17/19)	Transported by ambulance for gastrointestinal illness	RCT	Bilateral auricular acupressure (NR)	Relaxation	(1) Sham acupressure	VAS (.002)	NR

Agarwal et al. (2005, India) [8]	18–50	76 (36/36)	Elective surgical procedure	RCT	Acupressure (10 min, 20–25 cyc/min manually rotated)	Yin Tang	(1) Sham acupressure	VSS (<.001)	NR

Borimnejad et al. (2012, China) [16]	9–12	80 (40/40)	Elective surgery	RCT	Acupressure (30 min, 1.3 psi acupressure bead)	Yin Tang	(1) Sham acupressure	STAIC (NS^j^)	NR

Valiee et al. (2012, Iran) [17]	44.04 ± 11.25 (mean age)	70 (35/35)	Abdominal surgery (cholecystectomy, hysterectomy, herniorrhaphy, laparoscopy)	RCT	Acupressure (10 min, nondominant, 20–25 cyc/min, acupressure bead) Auricular acupressure	Shenmen Yin Tang	(1) Sham acupressure	VAS (<.001)	NR

^a^Acu: acupuncture; ^b^Sham: sham acupuncture; ^c^Con: control group; ^d^RCT: randomized controlled trials; ^e^STAI: the State-Trait Anxiety Inventory; ^f^NR: not reported; ^g^PONV: postoperative nausea and vomiting; ^h^VSS: verbal-scale score; ^i^VAS: visual analogue scale; ^j^NS: not significant; ^k^LI: large intestine; ^l^LV: liver; ^m^PC: pericardium; ^n^HT: heart; ^o^TE: triple energizer; ^p^LU: lung.
